# Enhanced Production and Characterization of a Highly Stable Extracellular Protease from an Extreme Halophilic Isolate *Salicola marasensis *

**Published:** 2018

**Authors:** Nika Khoshnevis, Shahla Rezaei, Amene Samaei-Nouroozi, Mohsen Amin, Mahsa Moshfegh, Mohammad Reza Khoshayand, Mohammad Ali Faramarzi

**Affiliations:** a *Department of Pharmaceutical Biotechnology, Faculty of Pharmacy and Biotechnology Research Center, Tehran University of Medical Sciences, Tehran, Iran. *; b *Department of Drug and Food Control, Faculty of Pharmacy and Pharmaceuticals Quality Assurance Research Center, Tehran University of Medical Sciences, Tehran, Iran.*

**Keywords:** Salicola marasensis, Halophile, Proteolytic extract, Protease activity, Optimization, Characterization

## Abstract

Owing to their superior catalytic activity in the extreme conditions, extremozymes have found the potential biotechnological applications for industrial purposes. A robust extracellular protease activity was detected in the culture broth of *Salicola marasensis*, an extreme halophilic bacterium, after a 48 h-incubation. The effect of different media ingredients in a liquid state fermentation was followed with the aim of improving the enzyme production yield. Fractional factorial and Box-Behnken designs were applied to get a 3.4 fold (from 6.0 to 20.3 U mL^−1^) improvement of protease production. The distinguishing features of this enzyme were stability at a wide range of pH (5.0–11.0) and temperature (25–60 °C), significant compatibility towards organic solvents, metal ions, chemicals, and surfactants, and hydrolysis of a variety of substrates. The properties of this enzyme can be of tremendous help in terms of the halophilic proteolytic extract’s industrial applications.

## Introduction

A continuous stream of needs to innovative products has driven demand for industrial enzymes especially hydrolytic enzymes such as proteases as the heart of industrial biotechnology. With this ever-increasing demand, use of extremozymes has gained notable attention during the last several years in many industrial processes being carried out under many harsh conditions (1, 2). Adjustment of industrial harsh conditions to standard values needed for activity and stability of the available enzymes cannot always be possible. Therefore, it would be critical to have available enzymes showing optimal activities at extreme values of pH, temperature, and varied concentrations of salts, organic solvents, surfactants, and chemicals (3, 4). Recently, great interest has been drawn on enzymes of halophilic microorganisms and their biotechnological potentials. Halophiles are excellent sources of enzymes that are not only salt stable but also can withstand and carry out reactions efficiently under different extreme conditions (5-7).

Among the enzymes with commercial use, alkaline or serine proteases sourced from bacteria as the principal class of microbial proteases, have wide-application spectra and novel properties due to their exotic catalytic natures (8, 9). These enzymes in industry occupy a pivotal position because of their wide applications and hold a major share of the enzyme market with two-thirds of the total worldwide sale of enzymes (8). With arriving new frontiers in bio-industry, the spectrum of protease applications has expanded into many new fields (8−11).

Halophilic proteases are adapted to extreme environments, and are unusually stable. This property makes them suitable candidates for catalyzing under harsh conditions such as high temperatures, pH values, and ionic strength and in the presence of high concentrations of organic solvents, surfactants, metal ions, *etc.* (12−14). So far, proteases from halophiles have found wide applications in the food, leather, and detergents industries; the biodegradation of pollutants in salty habitats; and enzymatic synthesis in non-aqueous media (12, 14). Given the present and the next applications of halophilic proteases, the search for new promising halophilic protease-producers with raised stability is a continuing process aimed at improving their adoption in special processes.

The current study aimed to isolate a halophilic strain capable of producing protease, identified by morphological and biochemical characteristics followed by 16s rRNA analysis. In this investigation, statistical and non-statistical strategies were applied to optimize media composition and physical limits for the maximum production of the alkaline protease using a liquid fermentation. The enzyme was then concentrated and characteristics of the concentrated extracellular protease, especially its activity and stability in the presence of salts, organic solvents, metal ions, surfactants, and inhibitors were determined.

## Experimental

*Chemicals*

Skimmed milk, Folin-Ciocalteu reagent (FCR), culture media and buffer components, and also surfactants were purchased from Merck (Darmstadt, Germany). Casein, phenylmethylsulfonyl fluoride (PMSF), and tyrosine were provided from Sigma-Aldrich (St. Louis, MO, USA). Other chemicals were of analytical grade and were commercially available.

*The screening of halophilic bacteria for the extracellular protease activity and identification of the selected strain*

To isolate the halophilic bacteria, the samples were collected aseptically from different salty districts of Iran. Isolation of halophilic bacteria using salt enrichment was done using the modified Luria-Bertani (LB) agar medium containing (g L^-1^ distilled water): yeast extract, 5; peptone, 10; agar, 15; and NaCl, 200. pH of the medium was adjusted at 7.5 and sterilized by autoclaving (121 °C and 15 psi for 20 min). Afterwards, 1 g soil was suspended in 1 mL sterile phosphate buffer saline (PBS) containing 20% NaCl, and after vortexing, 500 μL of suspension (or water samples) was spread over the LB agar plates. After incubating at 37 °C for 48 h, isolation was completed by serial dilution and plated on the LB agar medium. The colonies were often streaked on the LB agar medium for purification and stored on the modified agar plates at 4 °C. Stock cultures of the pure bacterial colonies were maintained as a glycerol stock (the modified LB broth medium containing 20% w/v NaCl and 15% v/v glycerol) at −85 °C.

To screen the protease-producing colonies, the purified bacteria were transferred to the skimmed milk agar plates (1% (w/v) skimmed milk powder and 20% (w/v) NaCl). The colonies with the proteolytic activity showed clear zone. The isolated strains with the maximal protease activity, as measured by the ratio of hydrolytic zone diameter (mm) to the colony diameter (mm), was subjected to screen for the protease production in the modified LB broth medium (containing 20% NaCl). The isolate with the highest proteolytic activity in the modified LB broth medium was subjected for subsequent studies.

To identify the strain, the selected pure strain was streaked on the modified LB agar plate as described above and incubated for 48 h at 37 °C. The applied morphological tests were gram staining, checking the shape and color of the colony, and spore-forming ability. The biochemical and physiological characteristics were further determined using a LB 48 h-old culture of the purified isolate and included catalase and oxidase, motility, Voges-Proskauer, and methyl red tests, growth at different temperatures, pH values, and NaCl concentrations, the fermentation of some sugars, hydrolysis of starch, gelatine, casein, Tween 80, nitrate reduction, and H_2_S and indole production. For molecular characterization, the bacterial genomic DNA of the isolate was extracted by Genomic DNA Extraction Kit (Bioneer, Korea) under the manufacturer’s protocol. A pair of 16S rRNA universal primers, including 27F as a forward primer (5′-AGAGTTTGATCCTGGCTCAG-3′) and 1525R as a reverse primer (5́ -TTCCTCCACTAGGTCGG-3′) (15), were used to amplify a ~1.5 kb fragment of 16S rRNA gene using polymerase chain reaction (PCR) was performed in a reaction volume of 100 µL containing PCR buffer with 1.5 mM MgCl_2_, 200 µM dNTPs, Taq DNA polymerase (5.0 U), 1.0 µM each primer, and DNA template (50 ng). The PCR amplification was programmed as follows: (i) initial denaturation at 94 °C for 5 min; (ii) 30 cycles of denaturation at 94 °C for 1 min, annealing at 56 ºC for 1 min and extension at 72 ºC for 1.5 min; and (iii) final elongation at 72 °C for 10 min. Following the visualization of the amplified DNA using gel electrophoresis, DNA fragment of ~ 1.4 kb was eluted from gel using a QIAGEN gel extraction kit. The extracted fragment was employed as a template for amplifying forward and reverse strands using the same primers, and separate sequencing of the PCR products. The phylogenetic neighbors were determined by BLASTn (NCBI, http://ncbi.nlm.nih.gov/BLAST/) against GenBank^® ^database at the NCBI server. As a result, strain SR-081 Halo (sampled from Golestan Salt Lake, Iran (37°24′3′′N 54°38′44′′E)) was designated as *Salicola marasensis* (*S. marasensis*) and deposited in NCBI with the accession number of KF859984 (http//www.ncbi.nlm.nih.org/ available Jan 18, 2018). Then, the 16S rDNA sequences of the close relatives of the isolate were retrieved from the GenBank^® ^database and the phylogenetic tree was constructed by the neighbor-joining method. The significance of the junctions was stablished using the bootstrap method (1000 replicates).

*Media, growth behavior, and the production of* *proteolytic extract by S. marasensis*

Growth and protease production of *S. marasensis *were studied in the minimal basal medium (16) consisting of (g L^−1^): yeast extract, 5; MgCl_2_.6H_2_O, 20; K_2_SO_4_, 5; CaCl_2_.2H_2_O, 0.1; and NaCl, 200. Fifty mL of the basal medium were taken in 250 mL Erlenmeyer flasks and pH was adjusted to 7.4 before being autoclaved at 121 °C and 15 psi for 20 min. The flasks were inoculated with 1 mL of a two-day-old mother culture of *S. marasensis* (OD_600_ ~ 1) and incubated at 37 °C with continuous shaking at 150 rpm for 120 h. The samples were withdrawn periodically at the 24-h intervals and analyzed for the protease production and the growth estimation. Bacterial growth was estimated by the cell number determination. To oversee the proteolytic activity, the withdrawn samples were centrifuged at 8,000×g for 10 min and the cell-free supernatant was assayed for the extracellular proteolytic activity.

*Protease assay and protein determination*

Proteolytic activity was determined toward casein following the method described by Kunitz and Gen (17) with some modifications. Briefly, 0.5 mL of casein (0.5% (w/v) prepared in 50 mM phosphate buffer, pH 8.0, containing 20% (w/v) NaCl was added to 1 mL (~ 5 U) of the enzyme sample to start the reaction. The mixture was immediately incubated at 40 °C for 15 min, and the reaction was stopped by adding 0.5 mL of 60% TCA. After 5 min standing at room temperature, the precipitate was centrifuged at 18,000×g for 10 min to be removed and the supernatant was used to estimate the free tyrosine amount. One mL of Na_2_CO_3_ (0.5 M, to naturalize the supernatant) and 250 µL of the FCR (1 N) were added to 0.5 mL of the supernatant. Followed by keeping the mixture at 37 °C for 15 min, the absorbance was recorded with an UV/Vis spectrophotometer at 660 nm against the corresponding blank. As a blank, the enzyme was inactivated at the beginning of the incubation period. The proteolytic activity of the samples was measured based on the tyrosine standard curve, and one unit of proteolytic activity was expressed as the enzyme quantity, releasing 1 µmol of tyrosine min^−1^ mL^−1^ under the standard assay conditions. The standard curve was made by 0–1000 µmol mL^−1^ of tyrosine. All analyses were carried out in triplicate; the results were taken in the means of three independent determinations. In addition, the protein concentration was determined by the dye binding assay suggested by Bradford (18), using bovine serum albumin (BSA) to prepare the standard solution range.

*Medium optimization for maximum protease production by S. marasensis*

*Preliminary study*

Considering that the broad applications of halophilic proteases demand efficient production, one-variable-at-a-time strategy was applied to early screen of the most significant items needed to increase the growth and proteolytic activity. Therefore, the conditional tests, including temperature (25–45 °C), pH (5.0–10.0), inoculum size (1–10%), agitation speed (50–150 rpm), fermentation period (24–120 h), and aeration (40–90%, according to the total volume of Erlenmeyer flask occupied by air) were investigated. To find the most effective nutritional sources on the growth and proteolytic activity, several carbon sources (starch, fructose, sucrose, lactose, glucose, and maltose), nitrogen sources (yeast extract, peptone, glycine, gelatin, ammonium oxalate, potassium nitrate, and skimmed milk powder), trace elements (MgCl_2_.6H_2_O, K_2_SO_4_, CaCl_2_.2H_2_O, MnSO_4_, FeCl_2_, KNO_3_, trisodium citrate, and Na_2_CO_3_), and salts (NaCl, KCl, and LiCl) were implemented. For evaluating the effects of the nitrogen sources, the additive sources were applied at 0.5% (w/v) concentration in addition to 0.5% (w/v) yeast extract already present in the basal medium. Also all the carbon sources were added at 1% (w/v) level. The effect of a variety of inorganic elements on the proteolytic activity also was checked using individually removal of MgCl_2_.6H_2_O, K_2_SO_4_, and CaCl_2_.2H_2_O from the basal medium and individually addition of MnSO_4_, FeCl_2_, KNO_3_, trisodium citrate, and Na_2_CO_3 _to the basal medium at the final concentration of 0.05, 0.005, and 0.5% (w/v), respectively. Similarly, the effect of salts (NaCl, KCl, and LiCl at the concentrations of 0–30%) on the strain growth and proteolytic activity was evaluated.

*Experimental design*

The screening design: Taking the above factors with an effect on the proteolytic activity in consideration, the fractional factorial design (FFD) was adopted to identify the items having the most significant effects. In this design, a resolution III 2^(7-4)^ FFD was employed to reach the main effects of each factor and clarification of two-factor interactions. The effect of seven factors on the proteolytic activity at two levels (g L^−1^) (based on the literature) of low (−1) and high (+1) were investigated after 48 h of incubation at 40 ºC and pH 7.0. The levels were included peptone (X_1_; 0.1–1.0), glucose (X_2_; 0.5–2.0), MgCl_2_.6H_2_O (X_3_; 0.1–1.0), K_2_SO_4_ (X_4_; 0.1–1.0), CaCl_2_.2H_2_O (X_5_; 0.05–0.50), KNO_3_ (X_6_; 0.1–1.0), and MnSO_4_ (X_7_; 0.05–0.25). The matrix covering 11 experiments (3 were center point runs to show lack of fit) has been shown in Table 1. The design was made and analyzed using statistical software package, Design-Expert (version 7.0.0; Stat-Ease; Inc., Minneapolis, Minnesota, USA). The model equation for 2^7-4^ design was:

Y = β_0_ + β_1_ X_1_ + β_2_ X_2_ + … +β_7_ X_7_

where Y is the predicted response (proteolytic activity), β_0_ is the intercept, β_1_ is the coefficient for X_1_, and X_1_ is the first independent variable that explains the variance in *Y*. Statistical analysis of data with the aid of half-normal percentage probability plot and evaluation by analysis of variance (ANOVA) calculated *p-*value. Factors with *p*-value below 0.05 were considered to influence the proteolytic activity significantly and the model terms were selected or rejected based on the significance. All the experiments were performed in triplicate and the average of responses was attended.

The optimization design: The selected significant elements from the preliminary screening study and FFD were subjected to the Box-Behnken design (BBD) (19, 20) to optimize the proteolytic activity of *S. marasensis*. Based on the screening results, peptone (X_1_), K_2_SO_4_ (X_4_), and KNO_3_ (X_6_) were fed to the Box-Behnken design to be optimized and each variable was evaluated at three experimental levels coded as −1, 0, and +1. The variables were checked at three levels (g L^−1^): X_1_ (−1: 1.0; 0: 3.0, and +1: 5.0), X_4_ (−1: 1.0, 0: 3.0, and +1: 5.0), and X_6_ (−1: 1.0, 0: 5.5, and +1: 10.0). All the variables were taken at a central coded value considered as zero. As a result, a total of 17 trials, including 12 factorial points and 5 repeats at the center point for estimation of the pure error sum of squares were performed (Table 2). This design was applied using the Design-Expert® software. On completion of runs, the average maximal proteolytic activity yield was taken as the response (Y) and a second order polynomial equation was then fitted to the data by multiple regression procedure. For three-factors, a second-order polynomial equation that predicted the proteolytic activity was in the following form:

Y = β_0_ + β_1_ X_1_ + β_2_ X_2_ + β_3_ X_3_ + β_12_ X_1_ X_2_ + β_13_ X_1_ X_3_ + β_23_ X_2_ X_3_ + β_11_ X_1_^2^ + β_22_ X_2_^2^ + β_33_ X_3_^2^

**Table 1:** Fractional factorial design (2^7-4^) Res III. The matrix (Trial No. 1 to 8) and center point experiments (Trial No. 9 to 11) with the corresponding experimental

**Trial NO.**	**Variables**							**Response (U mL** ^−1^ **)**
	**X**_1_	**X**_2_	**X**_3_	**X**_4_	**X**_5_	**X**_6_	**X**_7_	
1	−1	−1	−1	+1	+1	+1	−1	10.4 ± 0.3
2	+1	−1	−1	−1	−1	+1	+1	11.4 ± 0.4
3	−1	+1	−1	−1	+1	−1	+1	10.7 ± 0.3
4	+1	+1	−1	+1	−1	−1	−1	7.7 ± 0.3
5	−1	−1	+1	+1	−1	−1	+1	8.2 ± 0.2
6	+1	−1	+1	−1	+1	−1	−1	8.9 ± 0.2
7	−1	+1	+1	−1	−1	+1	−1	12.0 ± 0.6
8	+1	+1	+1	+1	+1	+1	+1	9.1 ± 0.3
9	0	0	0	0	0	0	0	9.5 ± 0.3
10	0	0	0	0	0	0	0	10.5 ± 0.4
11	0	0	0	0	0	0	0	10.9 ± 0.4

Results shown are the average of three independent experiments ± SD.

where Y is the predicted response, β_0 _is the intercept, β_1_-β_3_ are the regression coefficients, β_11_-β_33_ are the squared effects, and X_1_-X_3_ are the independent variables. The fitness of the second order polynomial equation was evaluated by multiple correlation coefficient (R^2^) and adjusted R^2^. The responses under different combinations defined by design were analyzed using ANOVA to estimate the statistical parameters. Then, the response surface plots were constructed to study the interaction among factors. To validate the response surface model and evaluate the precision of the model, a random set of the optimized checkpoints experiments was set up according to the conditions predicted by the model. The experiments were conducted both within and outside the design space.

**Table 2:** Factorial points (Trial 1 to 12) and Replicates (Trial 12 to 17) in the matrix of Box-Behnken design

**Trial NO.**	**Variable**			**Response (U mL** ^−1^ **)**
	**Peptone**	**K**_2_ **SO**_4_	**KNO**_3_	
1	−1	−1	0	13.9 ± 0.6
2	+1	−1	0	10.5 ± 0.5
3	−1	+1	0	13.7 + 0.6
4	+1	+1	0	16.0 ± 0.9
5	−1	0	−1	13.6 ± 0.7
6	+1	0	−1	15.1 ± 0.7
7	−1	0	+1	13.4 ± 0.7
8	+1	0	+1	16.7 ± 0.6
9	0	−1	−1	12.9 ± 0.7
10	0	+1	−1	15.7 ± 0.7
11	0	−1	+1	16.6 ± 0.3
12	0	+1	+1	16.2 ± 0.5
13	0	0	0	18.7 ± 0.8
14	0	0	0	19.9 ± 0.9
15	0	0	0	20.1 ± 0.9
16	0	0	0	19.5 ± 0.4
17	0	0	0	20.3 ± 0.8

Results shown are the average of three independent experiments ± SD.

*Concentrated proteolytic extract preparation*

The experiment steps of crude enzyme preparation were carried out at 4 °C. The cells were harvested by centrifugation of a 2-day-old culture of *S. marasensis* at 8000×g for 10 min. The precipitation of the cell-free supernatant was performed by gradually adding up to an 80 % (v/v) saturation pre-cold ethanol on to the media and stirring for 2 h. The precipitate was concentrated by centrifugation at 20,000×g for 15 min and suspended in a minimal volume of 50 mM phosphate buffer (pH 8.0) including 20% NaCl. The concentrated extract was dialyzed against same buffer overnight and the proteolytic activity was measured in the assay conditions.

*The biochemical and functional characterization of the proteolytic extract*

*The thermal- and pH-stability of the proteolytic*

The stability of the crude enzyme was investigated in a broad range of pH (3.0–11.0) and temperature (25–65 °C). The enzyme samples (~ 20 U) were adjusted to the desired pH values and were incubated at different temperatures. After an hour, the residual activity of the proteolytic extract was assayed according to the method described in the earlier section and the initial proteolytic activity before starting the pre-incubation was considered as 100%. The buffer systems for pH were citrate buffer (pH 3.0–5.5), phosphate buffer (pH 6.0–8.0), Tris-HCl buffer (pH 8.5–9.5), and glycine-NaOH buffer (pH 10.0–11.0) at 0.1 M concentration, while 20% NaCl (w/v) was incorporated into the used buffers for optimal activity.

*The effects of inorganic salts and metal ions on the proteolytic stability*

The enzyme sample (~ 20 U) was added to phosphate buffer (pH 8.0) containing NaCl, KCl, and LiCl with the final concentration of 0–30% in the mixture. After an hour incubating at room temperature and shaking at 150 rpm, the residual proteolytic activity assayed and the original enzyme activity existed before the incubation period was considered as 100%. The stability of the crude protease was also investigated in the presence of Fe^3+^, Fe^2+^, Ni^2+^, Co^2+^, Hg^2+^, Mn^2+^, Mg^2+^, Zn^2+^, Cu^2+^, Al^3+^, and Li^+ ^at the concentrations of 1, 5, and 10 mM dissolved in 100 mM phosphate buffer (pH 8.0) containing 20% NaCl. The residual activity was determined after an hour of incubation at room temperature with an orbital shaking at 150 rpm. The enzyme activity in the absence of any di- and tri-valent ions was taken as 100%.

*The effects of surfactants, inhibitors, and organic solvents on the proteolytic stability*

The effects of several surfactants (cetyltrimethylammonium bromide (CTAB) 

(1-10 mM), Triton X-100 (1-10 mM), SDS (5-20 mM), and Tween 80 (1-5 mM)), and denaturing agent of urea (at the same concentrations of CTAB and Triton X-100) in 0.1 M phosphate buffer (pH 8.0) containing 20% NaCl on the enzyme stability were also studied. In addition, the effects of protease inhibitors of PMSF (1-10 mM), EDTA (1-10 mM), and 2-ME (10-100 mM) were investigated. The mixture of protease extract (~ 20 U) and agents was incubated at room temperature with an orbital constant shaking at 150 rpm for an hour and the residual activity was assayed by the procedure. The proteolytic activity without any additives was set as 100%.

In addition, the stability of the crude protease in organic solvents with various log *P* values at the concentrations of 20, 50, and 80% (v/v) was studied. Organic solvents chosen in this study were acetonitrile, acetone, ethanol, butanol, hexanone, cyclohexanol, chloroform, octanol, cyclohexane, hexane, heptane, decanol, nonane, dodecane, and hexadecane. The mixture of protease extract (~ 20 U)-solvent was incubated at room temperature with a constant shaking at 150 rpm for an hour. The aqueous phase was carefully withdrawn and assayed and the residual specific proteolytic activity was applied to express the enzyme stability. Each result was compared with the specific activity of the corresponding control.

*The substrate specificity analysis*

The substrate specification was investigated by incubating the crude protease with several substrates including casein, azocasein, albumin, and gelatin. Each substrate was dissolved in 100 mM phosphate buffer (pH 8.0) containing 20% NaCl to a concentration of 1% w/v. Then, substrate solution was mixed with 1 mL of the supernatant containing protease (~ 20 U) to obtain a final volume solution pointed in the protease assay, previously. Indeed to test the proteolytic extract substrate specificity, the assay procedure explained above was followed except casein was replaced by the specific protein substrate and the released tyrosine was estimated.

*Statistical analyses*

To show the significant difference between groups, One-way ANOVA followed by Holm-Sidak multiple comparison test and two-way ANOVA were applied. Statistical significance was considered at *p *< 0.05.

## Results and Discussion

*The identification of the selected bacterium and the extracellular protease production*

**Figure 1:**
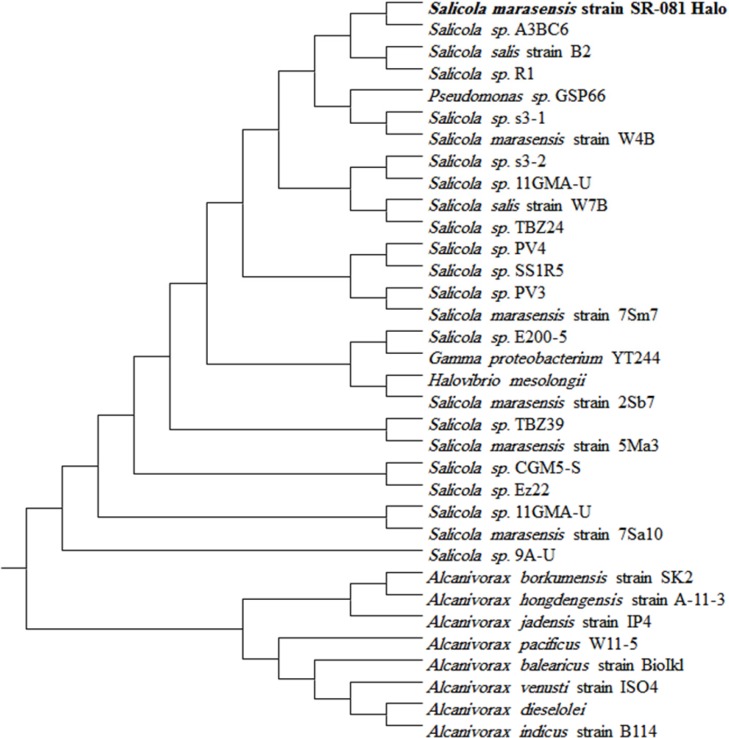
Tree showing the phylogenetic inter-relationships of *S. marasensis *and its closest relatives

**Figure 2:**
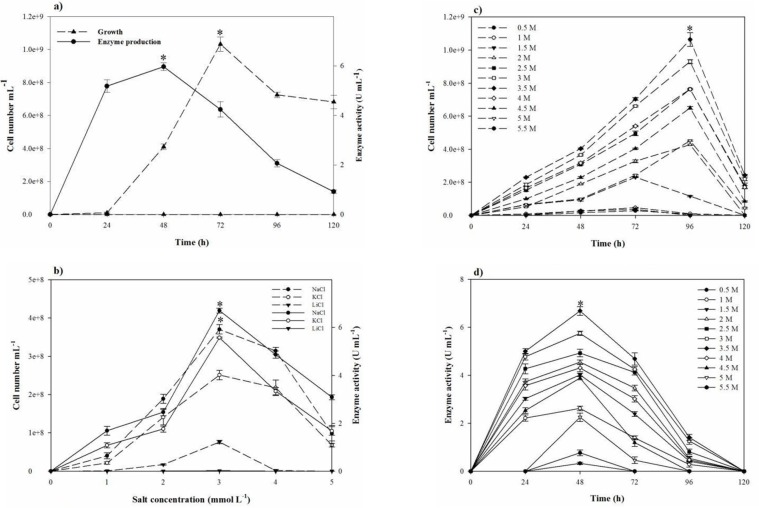
Growth behavior of *S. marasensis* and its keratinolytic protease production profile (a) over the time and (b) in the presence of a variety of salts after 48 h from incubation, (c, d) at a wide range of NaCl at the fermentation period of 120 h

Based on the clearance zone around the purified colonies on the skimmed milk agar plates, out of 60 halophilic isolates collected from different salty areas of Iran, six were selected with proteolytic activity. The selected isolates were subjected to further studies for deciding on a strain with the great proteolytic activity in the modified LB broth medium. Finally, strain SR-081 Halo showed the maximal proteolytic activity. Results of the morphological, biochemical, and physiological identifying studies of the selected strain are listed in Table 3. Analysis of the 16S rRNA sequence revealed its relatedness to *Salicola marasensis*. The 16S rRNA sequence (1417 bp) was submitted to GenBank^®^, assigned the accession number of KF859984, and designated as *Salicola marasensis *SR-081 Halo. To construct the *Salicola marasensis *SR-081 Halo phylogenetic tree, the 16S rRNA gene sequence analysis was performed with the NCBI dataset and finally, a consensus tree was drawn using the neighbor-joining algorithm (Figure 1). The growth and proteolytic activity of *Salicola marasensis *were studied in the basal medium for 120 h. The Maximal proteolytic activity (6 U mL^−1^) was found at 48 h of fermentation period, which coincided with the isolate high growth at the exponential phase (Figure 2a). The maximal enzyme production at the early and middle exponential phase suggested that the extracellular proteolytic content of *S. marasensis* is produced as a primary metabolite. A decline in the enzymatic activity was detected by increasing the fermentation period, when 4.3, 2.1, and 0.9 U mL^−1^ were recorded at 72, 96, and 120 h of fermentation. This decrease might be caused by auto-proteolysis or protease degradation by the other proteases, which were produced during the same growth phase. According to the literature, this is the first report on the *S. marasensis *proteolytic extract optimization and characterization.

**Table 3:** Physiological and biochemical characteristic of selected isolate SR-081 Halo

**Test**	**Result**	**Test**	**Result**
Morphology	Rod	pH	5.0−9.0 (7.5)
Pigmentation	Cream	Hydrolysis of:	
Gram reaction	Negative	Casein	Positive
Spore formation	Positive	Gelatin	Positive
NaCl range (% w/v)	0−30 (12)	Starch	Positive
Motility	Positive	Tween 80	Positive
Catalase test	Positive	Acid production from:	
Oxidase test	Positive	Glucose	Negative
Nitrate reduction	Positive	Galactose	Positive
Voges-Proskauer test	Negative	Fructose	Negative
Methyl red test	Negative	Lactose	Negative
H_2_S production	Negative	Sucrose	Negative
Urease	Negative	Glycerol	Negative
Temperature (°C)	25−50 (40)	Maltose	Positive

*The screening and optimization of the most effective factors on the proteolytic activity*

*One-facto-at-a-time*

**Figure 3:**
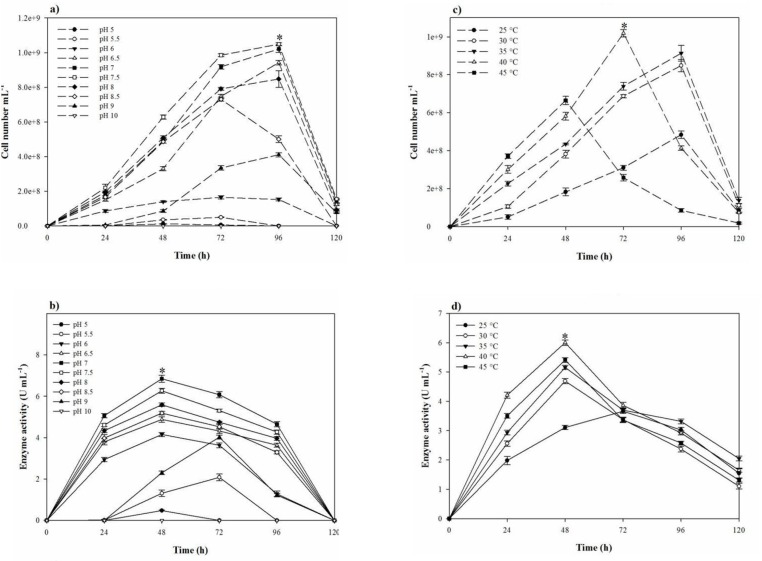
Growth of *S. marasensis* and its keratinolytic protease production profile (a, b) at a pH range of 5.0-10.0 and (c, d) at a temperature range of 25-45 °C

**Figure 4:**
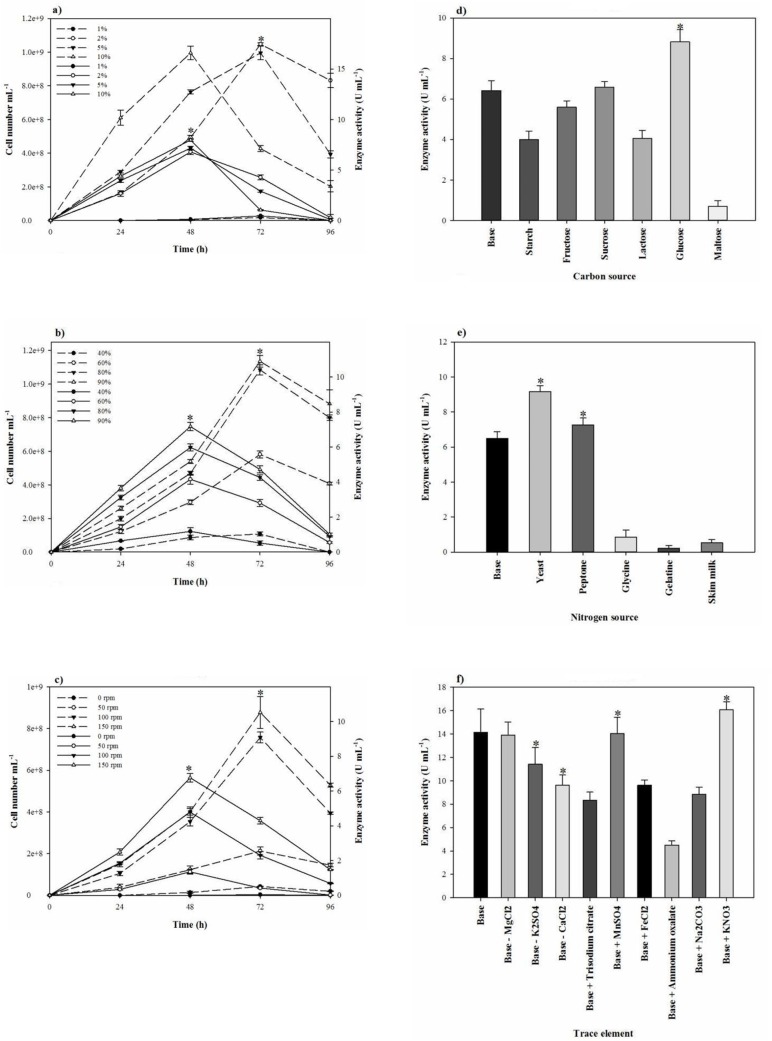
Effects of (a) aeration, (b) agitation rate, (c) inoculum density, (d) carbon sources, (e) nitrogen sources, and (f) trace elements on the strain growth and the proteolytic activity. Growth and enzyme production have been shown by a broken line and continues line, respectively

In the one-factor-at-a-time method, the most suitable salt requirement for the growth and the proteolytic activity (Figres 2b, 2c, and 2d) was found to be at 20% NaCl. Growth and the proteolytic activity of *S. marasensis* was strongly inhibited in the presence of LiCl. In addition, the maximal growth and proteolytic activity were occurred at pH 7.0-8.0 (Figures 3a and 3b), 40 °C (Figures 3c and 3d), and inoculum size of 10% (v/v) (Figure 4a).

**Figure 5:**
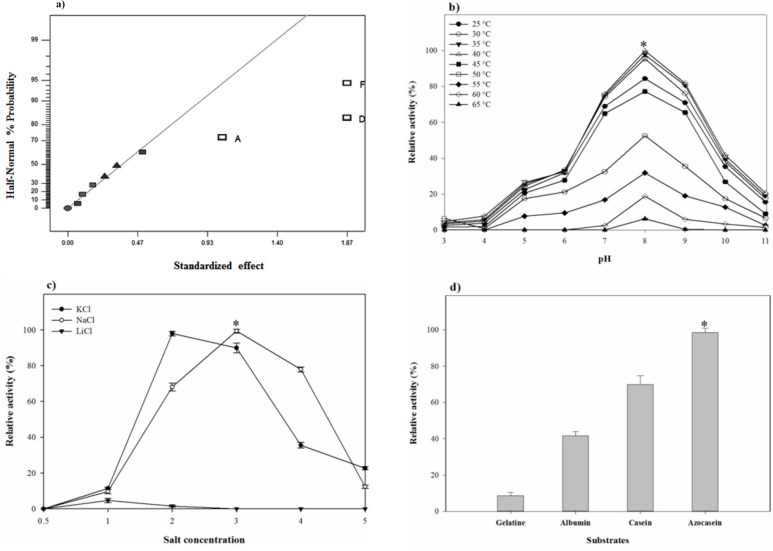
(a) Half-Normal plot for screening of most effective factors in Fractional Factorial design, effect of (b) pH-temperature and (c) different concentrations of NaCl, KCl, and LiCl on the proteolytic stability and (d) substrate specificity of *S. marasensis* proteolytic extract towards various substrates

To reduce the impact of the seed-starting medium on the composition of the fermentation medium and to prevent the rapid drop of proteolytic activity, 2% inoculum volume was selected. While the aeration and agitation rate of 90% and 150 rpm (Figures 4b and 4c), respectively, resulted in the maximal proteolytic activity, 80% aeration and agitation speed of 150 rpm were selected for subsequent experiments. The aeration and agitation rate affect the nutrient availability to the bacterium, thus, the isolate reaches to the stationary phase very soon and the maximal proteolytic activity take places in a minimal fermentation time. Glucose as the carbon source, yeast extract, KNO_3_, and peptone as the nitrogen sources, and alsoMgCl_2_.6H_2_O, K_2_SO_4_, CaCl_2_.2H_2_O, and MnSO_4_ as the trace elements were found to be that significant nutrition that increased the extracellular proteolytic content of the strain (Figure 5). Based on the results, these variables were selected for further statistical studies.

Since proteolytic activity and growth of *S. marasensis *SR-081 Halo were affected by the nutritional factors and the culture conditions, the medium was at first optimized by the one-factor-at-a-time method. At this study, lactose, maltose, and starch as carbon sources significantly repressed the proteolytic activity that may be the cause of catabolic repression. However, the production of proteolytic extract was promoted by glucose. The reason may be that heightened growth of the bacterium caused by glucose leads to the rich proteolytic activity formation and probably excretion of the proteolytic extract is directly related to the biomass production. Our results corroborate some of the previous reports showing the growth of *Streptococcus oralis *in a basal medium supplemented with glucose raised the proteolytic activity (21). On the contrary, glucose caused the reduction of the protease production in *Bacillus mojavensis* (22). The type of the used nitrogen sources also affected the enzyme production. Amongst the added nitrogen sources, yeast extract increased the proteolytic activity, followed by peptone. It seems bacteria prefer the more complex nitrogen sources that are metabolized slowly, probably from the fact that accumulations of free amino acids that may result from simple nitrogen sources repress the protease production. Glycine, gelatin, and skimmed milk as the sole nitrogen sources significantly reduced the extracellular proteolytic activity. There are few reports on the acting of the cited nitrogen sources as a protease promoter. According to the report of a protease from *Haloferax lucentensis *VKMM 007, gelatin increased the protease production (23). On the other hand, Sens *et al.* declared while peptone present in the media, smaller peptides along with them would be used because of easier substrate availability even without peptone hydrolyzing, resulting in the reduction of the protease production (24). This type of the protease reduction by increasing the peptone concentration also has been reported by Ferrero *et al.* (25) and Saurabh *et al.* (26). Another report from Karan and Singh (27) showed that the protease activity from *Geomicrobium *sp. EMB2 (MTCC 10310) in the presence of peptone is increased. KNO_3_ as an added trace element to the protease production medium significantly increased the yield of enzyme. It seems the enhanced proteolytic activity by KNO3 is not related to the nitrogen present in. This may be caused by potassium that plays an important role in refolding the partially unfolded protease, as K_2_SO_4_ stimulated the proteolytic activity and was subjected to subsequent experiments (28). In addition, the proteolytic activity was substantially improved when MgCl_2_.6H_2_O, MnSO_4_, and CaCl_2_.2H_2_O were used to supplement the medium. Mg^+2^ is essential not only during the growth, but similar to the other divalent cations such as Ca^+2^ and Mn^+2^ can involve in keeping the stability of the enzyme and protection against autolysis. These results corroborate the previous findings of metal ions strengthening the proteolytic activity (14, 25 and 29). Some reports showed conflicting results 

(30, 31).

*The fractional factorial design*

After the preliminary studies, peptone, glucose, KNO_3_, MgCl_2_.6H_2_O, K_2_SO_4_, CaCl_2_.2H_2_O, and MnSO_4_ were the main influencing factors on the proteolytic activity. Despite yeast extract was the most effective nitrogen source on the proteolytic activity, statistical studies showed its interference with the other media ingredients; therefore, peptone was used as a substituted.

**Figure 6:**
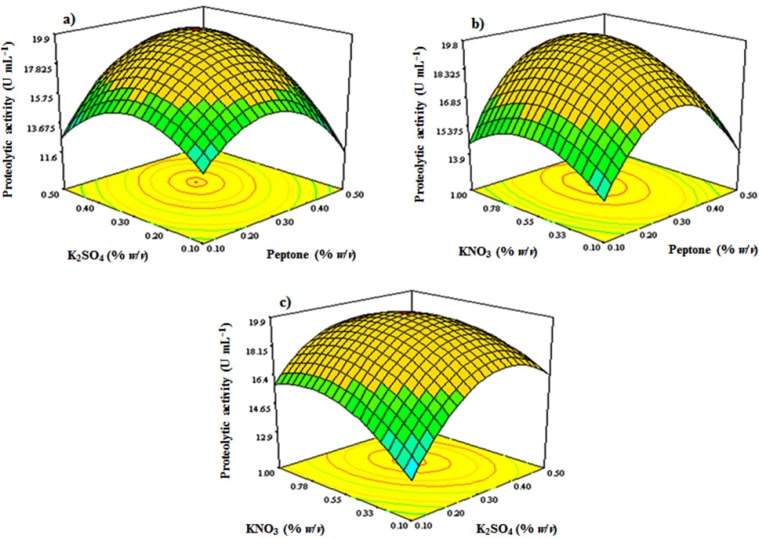
Response surface plots showing combined effects of (a) K_2_SO_4_ and peptone, (b) KNO_3_ and peptone, and (c) KNO_3_ and K_2_SO_4_ on *S. marasensis* proteolytic activity at the central values of all other parameters concentrations

For the early screening using the fractional factorial design, the effect of seven media elements and their interactions on the proteolytic activity of the isolate was investigated at 11 trials. In these experiments, 3 replicates were designed at center points according to the experimental matrix shown in Table 1 and the proteolytic activity was measured as the response. Experimental data from this model were analyzed and results from ANOVA with the aid of half-normal % probability plot were presented in Table 4 and Figure 6a. Out of seven variables studied, peptone (X_1_), K_2_SO_4_ (X_4_), and KNO_3 _(X_6_) had *p*-values < 0.05 based on ANOVA and were identified as the significant factors. The equation given below describes the relationships between the factors:

Y (proteolytic activity) = 10.43148 - 1.14815 X_1_ - 2.07407 X_4_ + 2.07404 X_6_

Multiple correlation coefficient (R^2^) of this first order model was 92%, which means that 92% of the data variation can be evaluated by the model. However, the difference between this value and predicted R^2^ (75%) revealed a first-order model is not an adequate mathematical equation for the demonstration of the relationship between the significant independent variables and the response. Therefore, a second-order model should be employed for further investigation.

**Table 4:** Analysis of variance (ANOVA results) for selected fractional factorial model

**Source**	**Sum of square**	**Df**	**Mean square**	***F-*** **value**	***p*** **-Value**
Model	16.1	3	5.4	21.1	0.0014
Peptone	2.1	1	2.1	8.4	0.0272
K_2_SO_4_	7.0	1	7.0	27.5	0.0019
KNO_3_	7.0	1	7.0	27.5	0.0019
Curvature	0.5	1	0.5	2.1	0.2015
Residual	1.5	6	0.3		
Lack of fit	0.6	4	0.2	0.3	0.8523
Pure Error	0.9	2	0.5		
Cor Total	18.1	10			
R^2^	0.9				
Predicted R^2^	0.8				

*The Box-Behnken design*

According to the results of FFD, factors of peptone, K_2_SO_4_, and KNO_3_ with high significance levels were chosen to be optimized using response surface methodology applying a 3-factors-3-levels Box-Behnken design. Box-Behnken design matrix included 5 central points and 12 axial points. Therefore, a group of 17 treatments with different combinations of peptone, K_2_SO_4_, and KNO_3_ were performed (Table 2). 

The significance of the model was evaluated by statistical analysis of data with *F-*value of 12.29 (Table 5). Further analysis of the data with stepwise regression revealed the relationship between the factors and the response as follows:

Y = 2.986 + 40.20 (X_1_) + 40.57422 (X_4_) + 11.24115 (X_6_) + 35 (X_1_. X_4_) + 5 (X_1_.X_6_) − 8.888 (X_4_. X_6_) − 85.125 (X_1_)^2^ − 68.875 (X_4_)^2^ − 7.76132 (X_6_)^2^

where Y is the proteolytic activity as the response and X_1_, X_4,_ and X_6_ are peptone, K_2_SO_4_ and KNO_3_ concentrations. Square correlation coefficient (R^2^) was determined to be 0.94, which implies that 94% of the variations in the proteolytic activity could be explained by the model. Also, the value of coefficient of variation (CV%) was 6.62, which shows a good precision and reliability of the experiments. 3D contour plots study the relationship between the independent variables and the response by which described the interaction of two variables while the third variable held at its middle level value. As is shown in Figure 6, the response surface visualized by 3D contour plots resulting from a second-order polynomial equation, shows an elliptical profile of remarkable interaction between peptone and KNO_3_, KNO_3 _and K_2_SO_4_, and K_2_SO_4 _and peptone, thus the best value for the response could be studied in the range of the variables. By analyzing and solving the results from the Box-Behnken model equation, a combination of adjusting peptone 3.0 g L^−1^, KNO_3_ 5.5 g L^−1^, and K_2_SO_4_ 3.0 g L^−1^ would reach to the maximal proteolytic activity yield of 20.3 U mL^−1^. Figure 6 shows the 3D contour plots of response surface.

To verify the model adequacy, the optimal conditions for the proteolytic yield were applied and the maximal response of the protease production was 19.93 U mL^−1^. The results confirmed the protease production optimizing by response surface methodology.

The final optimized conditions for extracellular proteolytic content of *S. marasensis *were found at (g L^−1^): NaCl , 200; glucose, 12.5; peptone, 3.0; MgCl_2_.6H_2_O, 5.5; CaCl_2_, 2.8; MnSO_4_, 1.5 g; K_2_SO_4_, 3.0; KNO_3_, 5.5; pH 7; 2% inoculum; and 40 °C. After optimizing the conditions, approximately 3.4-fold (from 6.0 to 20.3 U mL^−1^) enhancement of the proteolytic activity was achieved. Lately, the enzyme media optimization has been widely reported. The protease activity from *Lysinibacillus fusiformis *AU01 in the presence of 2.5 g L^−1^ of trisodium citrate, 3.6 g L^−1^ of yeast extract, pH 7.9, and 40 °C was increased 6 fold using the Plackett-Burman and Central Composite designs (31). Rai and Mukherjee (32) also reported a new alkaline serine protease from *Bacillus subtilis *DM-04 with highest activity at 45 °C, 72 h of incubation, and 1.25% (w/v) of beef extract as the most effective factors on the protease production.

**Table 5:** Analysis of variance (ANOVA results) for Response Surface Quadratic Model

**Source**	**Sum of Squares**	**df**	**Mean Square**	***F*** **-Value**	***p*** **-value Prob > F**
Model	125.0	9	13.9	12.3	0.0016
X_1_-peptone	1.8	1	1.8	1.6	0.2467
X_4_-K_2_SO_4_	7.5	1	7.5	6.6	0.0369
X_6_-KNO_3_	3.8	1	3.8	3.4	0.1083
X_1_X_4_	7.8	1	7.8	6.9	0.0337
X_1_X_6_	0.8	1	0.8	0.7	0.4251
X_4_X_6_	2.6	1	2.6	2.3	0.1760
X_1_^2	48.8	1	48.8	43.2	0.0003
X_4_^2	32.0	1	32.0	28.3	0.0011
X_6_^2	10.4	1	10.4	9.2	0.0190
Residual	7.9	7	1.1		
Lack of Fit	6.4	3	2.1	5.7	0.0629
Pure Error	1.5	4	0.4		
Cor Total	132.9	16			

*The functional and biochemical characterization of the* *proteolytic extract*

*The effect of pH and temperature on the proteolytic stability*

The pH-temperature stability of the crude protease was studied by evaluating the protease residual activity after an hour of incubation at different pH-temperatures. pH-temperature profile of the proteolytic extract is shown in Figure 5b. The proteolytic extract of *S. marasensis *was active over a wide range of pH from 4.0 to 10.0 and a temperature of 25-60 °C, with the maximal activity at pH 7.0–9.0 and 30–40 °C. The enzyme showed a high stability at the alkaline conditions and was retained higher than 75% of activity at 25–45 °C and pH 7.0–9.0. The residual activity less than 40% was remained at pH values below 6.0 and above 10.0 and temperatures higher than 55 °C. The pH-stability of the crude enzyme is comparable to the other haloalkaliphilic proteases reported in different studies as the maximal alkaline protease stability of *Botrytis cinerea *and *Bacillus mojavensis* was obtained at pH 8.0 and 7.0, respectively (22, 30). Similar to the obtained results, two alkaline proteases from *Lysinibacillus fusiformis* AU01 and *Halobacterium* sp. also showed maximal stability at 35-40 °C (14, 31). A protease from *Bacillus brevis* was also reported as a thermostable protease and showed the maximal stbility at 37 °C (29).

According to the obtained results, the protease from *S. marasensis* SR-081 Halo, which showed thermos-stability under elevated temperatures of 25-60 °C, can be placed among the thermostable enzymes. Meanwhile, it was stable in the pH range of 5.0–11.0, pointing out a pH-stable nature. The stable alkaline enzymes have received much attention because of their tremendous potentiality in industrial processes.

*The effects of metal ions, NaCl, KCl, and LiCl on the proteolytic stability*

As shown in Figure 5c, the maximal proteolytic extract stability occurred at 18% NaCl and 12% KCl. The peak of proteolytic stability occurred at 18% NaCl and a decrease in salt content caused a sharp decline in the proteolytic stability resulting in 11.5% activity at 6% NaCl. On the contrary, NaCl concentrations higher than 18% caused a gradual loss in the proteolytic stability, in which the enzyme retained 40% of its maximal activity at 24% NaCl. The maximal stability of the proteolytic extract with KCl was at the concentration of 12% and there was a gradual decrease in its activity by increasing KCl concentrations from 18 to 30% (Figure 5c). It should be considered that by increasing NaCl content, the proteolytic activity against casein decreases because of conformation changes in casein, which makes the substrate unavailable as a target of protease (33). Sinha and Khare (12) studied the effect of salinity on the secondary or tertiary structure of the protease from *Virgibacillus *sp. EMB13 using the UV-CD spectra. They found that β sheet content of the protease was less in the absence of NaCl, and the halophilic enzymes required the presence of NaCl or KCl for their natural structure and maximal activity.

The results of the proteolytic extract stability against metal ions are described in Table 6. The stability studies using different concentrations of metal ions showed a pronounced effect on the proteolytic stability. Based on the results, adding Co^2+^ and Mn^2+^ raised the proteolytic stability, in which increased activities of 164 and 107% were achieved at 1mM concentration, respectively. The proteolytic extract in the present of the other tested di-valent cations*, i.e*. Fe^2+^, Zn^2+^, Cu^2+^, and Mg^2+^ up to 5 mM concentration was interestingly stable; as Fe^2+^, Zn^2+^, Cu^2+^, and Mg^2+^ concentration increased over 5 mM, the proteolytic activity decreased. Based on the results, the proteolytic activity was completely inhibited by all concentrations of Li^+^, Fe^3+^, Ni^2+^, and Al^3+^ resulted in 70% loss of activity at concentrations higher than 5 mM. Singh *et al.* (34) reported a protease with tolerance to Co^2+^ and Ni^2+^ and sensitivity to Hg^+^. Comparing the several earlier reports (29, 31 and 35), the proteolytic extract of *S. marasensis* was significantly more stable in the presence of a variety of metal ions and can be considered as a metal ion resistant enzyme. The differences between various reports come from the characteristics of each strain.

**Table 6:** The effects of metal ions on the* S. marasensis *serine protease stability

**Metal ion**	**Residual activity (%)**		
	**1 mM**	**5 mM**	**10 mM**
None	100	100	100
Al^3+^	41.6 ± 0.7	26.5 ± 0.5	10.9 ± 0.4
Ca^2+^	155.3 ± 1.5	121.5 ± 1.1	104.1 ± 1.1
Co^2+^	164.7 ± 1.6	111.5 ± 1.6	44.3 ± 0.8
Cu^2+^	83.1 ± 1.6	75.0 ± 0.9	3.7 ± 0.1
Fe^2+^	99.4 ± 1.2	87.6 ± 1.5	3.7 ± 0.2
Fe^3+^	49.2 ± 0.8	32.6 ± 0.6	6.9 ± 0.2
Hg^2+^	65.5 ± 1.2	8.4 ± 0.3	1.6 ± 0.1
Li^+^	0	0	0
Mg^2+^	67.9 ± 0.8	59.2 ± 0.9	10.7 ± 0.3
Mn^2+^	107.6 ± 1.5	89.5 ± 1.3	4.4 ± 0.2
Ni^2+^	47.6 ± 1	33.9 ± 0.2	1.4 ± 0.1
Zn^2+^	83.0 ± 1	60.7 ± .68	1.6 ± 0.1

Results shown are the average of three independent experiments ± SD.

*The effects of detergents, chemicals, and inhibitors on the proteolytic stability*

The effects of several detergents, chemicals, and inhibitors on the proteolytic stability are summarized in Table 7. The proteolytic stability was increased interestingly in the presence of 2-ME (10 mM) and DTT (5 mM) more than 2- and 3-fold, respectively, suggesting 2-ME and DTT may be by maintaining free sulfhydryl residues in reduced form, improving the proteolytic activity (36). The partial inhibition by 2-ME and DTT at high concentrations might be due to the reduction of intramolecular disulfide bonds required to keep the activity and stability of the enzyme. A slight increase of the stability was observed in the presence of EDTA and CTAB as a cationic surfactant at low concentration (1 mM). This improvement probably is caused by the removal of interfering ions and neutralization of negative charges that are presented in plentiful on the surface of the halophilic proteins. EDTA and CTAB at higher concentrations had no significant effects on the proteolytic enzyme stability. The proteolytic extract retained its original stability in the presence of EDTA, showing that metallic ions are not necessary for the observed proteolytic stability and the proteolytic activity is not related to metalloproteases (36). On the other hand, decrease in the proteolytic stability at high concentrations of EDTA may be due to chelating of the present useful cations for the proteolytic stability. By adding 10 mM PMSF as a serine protease inhibitor, the proteolytic activity was significantly reduced and confirmed the major protease of *S. marasensis *as a serine protease. It was interesting to note that SDS, Tween 80, and Triton X-100 at concentrations up to 5, 5, and 10 mM, respectively, did not significantly affect the present proteolytic stability. Similar effects of Tween 80, TritonX-100, and CTAB on the proteolytic stability were found in *Bacillus *sp. EMB9 (30). The effect of a variety of chemical agents on the proteolytic stability has been largely described previously.

**Table 7:** Influence of detergents, chemicals, and inhibitors on of the protease stability from *S. marasensis*

**Chemical agent**	**Residual activity (%)**
None	100
EDTA 1 mM	117.4 ± 1.1
EDTA 5 mM	72.1 ± 1.8
EDTA 10 mM	31.9 ± 0.5
PMSF 1mM	81.7 ± 1.2
PMSF 5 mM	56.9 ± 1.2
PMSF 10 mM	5.7 ± 0.6
2- ME 10 mM	221.4 ± 2.8
2- ME 50 mM	99.8 ± 1.2
2- ME 100 mM	73.3 ± 1.4
1,4-DTT 1 mM	198 ± 2.5
1,4-DTT 5 mM	374.2 ± 4.3
1,4-DTT 10 mM	89.6 ± 1.1
Urea 1 mM	87.2 ± 0.5
Urea 5 mM	49.4 ± 0.8
Urea 10 mM	5.6 ± 0.2
Triton X-100, 1 mM	87.0 ± 1.7
Triton X-100, 5 mM	77.7 ± 1.5
Triton X-100, 10 mM	63.5 ± 1.1
Tween 80, 1 mM	96.5 ± 1.0
Tween 80, 2 mM	83.4 ± 1.2
Tween 80, 5 mM	67.3 ± 1.3
CTAB 1 Mm	106.8 ± 1.2
CTAB 5 Mm	99.6 ± 1.3
CTAB 10 Mm	91.4 ± 1.6
SDS 5 mM	70.5 ± 1.1
SDS 10 mM	39.2 ± 0.6
SDS 20 mM	20.6 ± 0.3

Results shown are the average of three independent experiments ± SD.

The halophilic enzymes are tending to work under low water conditions and they assumed to tolerate the organic solvents. The present protease was stable against a wide spectrum of organic solvents with log *P *in the range of −0.24 to 8.80 (Table 8). Heptane, octanol, cyclohexanol, decanol, and cyclohexane (at 20 % v/v) had no significant effects on the proteolytic stability. In the presence of ethanol and hexanone, the enzymatic activity was steeply decreased. By adding the other solvents, in particular acetone, butanol, hexane, hexadecane, and decanol, a great improvement of the proteolytic stability even with high levels was achieved. In addition, stability of the enzyme in IL increased at two lower concentrations. There is a suggestion on the enzymes stability in organic solvents because of high kinetic barrier, which protect enzymes from conformation transition. In general, hydrophilic solvents are usually incompatible with the enzymatic stability, whereas the lipophilic solvents retain a high catalytic activity of the enzyme. Hydrophobic solvents and ILs do not separate critical bound water from the surface of the enzyme leading to unfolding the enzyme and cause the enzyme to be less stable (37). For the present enzyme, a great stability against either water-miscible or -immiscible solvents was observed and there was not a certain relationship between log *P* of the organic solvents and their effect on the enzyme stability (12, 33). Sinha and Khare (38) also reported exposing the dialyzed enzyme to organic solvents and recording a 90% loss of activity, implying the salt mediation between organic solvents and the enzyme stability. Similar organic solvent stability has been reported in *Streptomyces clavuligerus *strain Mit-1, which the produced proteolytic extract was more stable against organic solvents with higher log *P* (39). On the contrary, Sinsuwan *et al*. (40) reported a protease that was more stable against polar organic solvents. The effect of organic solvents on the activity and stability of enzymes seems to depend on the properties of the solvents and enzyme. The halophilic enzymes obtain ability to work at low water content like non-aqueous media and their stability increased in polar solvents, whereas polar solvents are generally known to inactivate the majority of the enzymes. This property is noteworthy especially when the amino acids used in the enzymatic peptide synthesis that should be performed in non-aqueous media, are solubilized in polar organic solvents (38). Indeed, stability of the proteolytic extract of *S. marasensis* SR-081 Halo in polar solvents makes the enzyme extract more useful by facilitating synthesis of the peptides in non-aqueous system.

**Table 8:** Compatibility of the alkaline protease from *S. marasensis* with a variety of organic solvents at three different concentrations

**Organic solvent**	**Log ** ***P***	**Residual specific activity (%)**		
		**20 (%)**	**50 (%)**	**80 (%)**
None	-	100	100	100
[Bmim][PF_6_]	−2.4	120.3 ± 1.9	62.1 ± 0.6	20.7 ± 0.1
Ethanol	−0.24	36.0 ± 1.2	32.0 ± 1.5	21.0 ± 0.4
Acetone	−0.24	193.0 ± 1.8	166.0 ± 1.7	141.0 ± 1.4
Butanol	0.8	270.9 ± 2.9	110.0 ± 1.8	93.0 ± 1.7
Hexanone	0.96	12.0 ± 0.6	6.0 ± 0.3	0.2 ± 0.0
Cyclohexanol	1.5	107.2 ± 1.5	41.5 ± 0.6	14.8 ± 0.4
Chloroform	2	159.2 ± 2.2	61.0 ± 0.9	17.4 ± 0.3
Octanol	2.9	77.6 ± 1.7	27.5 ± 0.8	3.6 ± 0.2
Cyclohexane	3.2	116.8 ± 0.6	53.0 ± 0.7	17.4 ± 0.2
Hexane	3.5	210.4 ± 1.6	119.5 ± 1.4	19.2 ± 0.4
Heptane	4	176.0 ± 0.9	39.5 ± 0.5	14.0 ± 0.3
Decanol	4.9	89.6 ± 1.0	47.0 ± 0.7	16.2 ± 0.3
Nonane	5.1	141.6 ± 1.3	78.5 ± 0.6	24.2 ± 0.3
Dodecane	6.6	169.6 ± 1.0	82.0 ± 0.6	3.6 ± 0.4
Hexadecane	8.8	228.0 ± 1.9	63.5 ± 0.9	15.6 ± 0.3

Results shown are the average of three independent experiments ± SD.

*The substrate specification*

Generally, the alkaline proteases hydrolyze a variety of substrates and have wide substrate specificity. The proteolytic extract of *S. marasensis *SR-081 Halo was found to degrade the most preferred substrate followed by azocasein (100%), casein (74%), albumin (43%), and gelatin (10%) (Figure 5d). This enzyme showed specificity towards azocasein as the more sensitive substrate when compared to all other tested substrates. An alkaline protease from *Lysinibacillus fusiformis *AU01 (31) and an organic solvent-stable serine protease from *Bacillus subtilis* DM-04 (32) showed highest activity toward azocasein followed by casein and gelatin. The substrate specificity pattern arises from the nature and specific structure of the enzyme-active site, which is not absolute and could be changed in different conditions.

## Conclusion

In the present study, a newly halophilic strain of *S. marasensis* capable of producing protease possessing a combination of several industrially important properties is reported. Optimizing of the culture media compositions led to 3.4-fold increase in the enzyme production, which means the productivity was greatly scalable. The activity in high content of both hydrophobic and hydrophilic organic solvents; stability in the presence of high concentrations of salts, surfactants, metal ions, and chemical agents; and retained high activity at high temperatures and over a versatile pH range make it potential candidate for the majority of the industrial processes, in which proteases are employed, such as detergent formulation, non-aqueous organic synthesis, bio-transformation, and bio-remediation. The secreted protease from *S. marasensis *also showed the broad range substrate specificity for both soluble and insoluble proteins. With all these distinguishing properties, further studies are merited to find out the related catalytic abilities of the produced protease in various harsh industrial 

conditions.
